# Does Discrimination Attenuate Age-Benefits in Emotional Health?

**DOI:** 10.1093/geroni/igaa057.1475

**Published:** 2020-12-16

**Authors:** Daisy Zavala, Elizabeth Munoz, Martin Sliwinski, Stacey Scott

**Affiliations:** 1 Stony Brook University, Nesconset, New York, United States; 2 The University of Texas at Austin, Austin, United States; 3 Penn State University, University Park, Pennsylvania, United States; 4 Stony Brook University, Stony Brook, New York, United States

## Abstract

Perceived discrimination has been found to negatively impact emotional health (e.g., depression, psychological distress). Few studies, however, have examined this association across adulthood. Strength and Vulnerability Integration Theory (SAVI) proposes that there may be age-benefits in emotion regulation, however these may be reduced when faced with stressors. We examined whether two forms of stress related to discrimination (i.e., subtle and major) moderated expected age-benefits in emotional outcomes. We predicted that individuals who experienced more discrimination would display worse emotional outcomes, and that this effect would be stronger with older age. Participants were 334 diverse adults (25-65 years, Mage = 47, 63% Female) from Bronx, New York. They reported major and subtle discrimination experiences, depressive symptoms, positive affect (PA), and negative affect (NA). Subtle discrimination was associated with greater NA and depressive symptoms and lower PA (ps <.0001), but the associations with depressive symptoms and PA did not vary by age. Age, however, moderated the relationship between subtle discrimination and NA (b = .022, t (22) = 2.04, p<.05). Specifically, the slope between subtle discrimination and NA was stronger with older age. Greater major discrimination was associated with greater NA and depressive symptoms (p<.05), but these effects were age invariant. These results demonstrate that subtle and major discrimination are associated with poorer emotional health across individuals. Partially consistent with SAVI, stressful experiences like subtle discrimination may serve as a boundary condition for age-benefits in emotional outcomes.

